# Dung Beetle Optimization with Deep Feature Fusion Model for Lung Cancer Detection and Classification

**DOI:** 10.3390/cancers15153982

**Published:** 2023-08-05

**Authors:** Mohammad Alamgeer, Nuha Alruwais, Haya Mesfer Alshahrani, Abdullah Mohamed, Mohammed Assiri

**Affiliations:** 1 Department of Information Systems, College of Science & Art at Mahayil, King Khalid University, Abha 61421, Saudi Arabia; 2 Department of Computer Science and Engineering, College of Applied Studies and Community Services, King Saud University, P.O. Box 22459, Riyadh 11495, Saudi Arabia; nalrowais@ksu.edu.sa; 3 Department of Information Systems, College of Computer and Information Sciences, Princess Nourah bint Abdulrahman University, P.O. Box 84428, Riyadh 11671, Saudi Arabia; hmalshahrani@pnu.edu.sa; 4 Research Centre, Future University in Egypt, New Cairo 11845, Egypt; mohamed.a@fue.edu.eg; 5 Department of Computer Science, College of Sciences and Humanities-Aflaj, Prince Sattam bin Abdulaziz University, Aflaj 16273, Saudi Arabia; m.assiri@psau.edu.sa

**Keywords:** lung cancer, deep learning, feature fusion model, dung beetle optimizer, computer-aided diagnosis

## Abstract

**Simple Summary:**

Medical imaging devices can be vital in primary-stage lung tumor analysis and the observation of lung tumors from the treatment. Many medical imaging modalities like computed tomography (CT), chest X-ray (CXR), molecular imaging, magnetic resonance imaging (MRI), and positron emission tomography (PET) systems are widely analyzed for lung cancer detection. This article presents a new dung beetle optimization modified deep feature fusion model for lung cancer detection and classification (DBOMDFF-LCC) technique.

**Abstract:**

Lung cancer is the main cause of cancer deaths all over the world. An important reason for these deaths was late analysis and worse prediction. With the accelerated improvement of deep learning (DL) approaches, DL can be effectively and widely executed for several real-world applications in healthcare systems, like medical image interpretation and disease analysis. Medical imaging devices can be vital in primary-stage lung tumor analysis and the observation of lung tumors from the treatment. Many medical imaging modalities like computed tomography (CT), chest X-ray (CXR), molecular imaging, magnetic resonance imaging (MRI), and positron emission tomography (PET) systems are widely analyzed for lung cancer detection. This article presents a new dung beetle optimization modified deep feature fusion model for lung cancer detection and classification (DBOMDFF-LCC) technique. The presented DBOMDFF-LCC technique mainly depends upon the feature fusion and hyperparameter tuning process. To accomplish this, the DBOMDFF-LCC technique uses a feature fusion process comprising three DL models, namely residual network (ResNet), densely connected network (DenseNet), and Inception-ResNet-v2. Furthermore, the DBO approach was employed for the optimum hyperparameter selection of three DL approaches. For lung cancer detection purposes, the DBOMDFF-LCC system utilizes a long short-term memory (LSTM) approach. The simulation result analysis of the DBOMDFF-LCC technique of the medical dataset is investigated using different evaluation metrics. The extensive comparative results highlighted the betterment of the DBOMDFF-LCC technique of lung cancer classification.

## 1. Introduction

Over the last few decades, lung cancer has been a major cause of mortality. One of the common symptoms of lung tumors is coughing, which requires special consideration because most of the patients who have a cough are smokers, the main group affected by chronic obstructive pulmonary disease, which itself causes coughing [1,2]. Thoracic computed tomography (CT) or chest X-rays (CXRs) are two common techniques for the diagnosis of lung tumors. Sometimes, positron emission tomography (PET) and magnetic resonance imaging (MRI) can be utilized during staging the size of cancer spreads, while CT and CXR assist to determine better therapeutic management [3]. Biopsy and bronchoscopy are necessary to provide information on the histological type and to define the actual diagnoses of lung tumors [4,5]. In earlier investigations, the occurrence of a benign tumor after a nodule discovery and diagnostic operation was proven to be as high as 40%, which highlights the importance of rigorous nodule screening before further invasive treatment to avoid unwanted complications or loss of pulmonary capacity and limit surgical risk [6].

Specific characteristics should be measured and recognized to identify malignant nodules [7,8]. Cancer probability can be assessed by using the recognized features and their fusion. But, this task can be highly challenging, even for medical experts, because nodule presence and positive cancer diagnoses are not simply interrelated [9]. A computer-aided diagnoses (CAD) approach uses earlier analyzed features that are in some way associated with cancer suspicion, like shape, sphericity, volume, subtlety, speculation, solidity, etc. They use Machine Learning (ML) systems such as Support Vector Machines (SVMs) to categorize the nodules as benign or malignant [10,11]. Although several studies use similar ML algorithms, the problem with this method is that for the system perform well, various parameters should be input on an individual basis for each case, thereby making it hard to reproduce proficient outcomes [12]. In addition, this makes the approach prone to variability among dissimilar screening parameters and different CT scans. The benefit of utilizing deep learning (DL) in CAD systems is that it could implement end-to-end recognition by learning one of the important features in a trained model [13,14]. This enables the network to work effectively when there is variation, as it captures nodule features in CT scans with different parameters [15]. When the network is trained, it can be predictable and capable of generalizing its learning and identifying malignant nodules in new cases.

This article presents a new dung beetle optimization modified deep feature fusion model for lung cancer detection and classification (DBOMDFF-LCC) technique. The presented DBOMDFF-LCC technique mainly depends upon the feature fusion and hyperparameter tuning process. To accomplish this, the DBOMDFF-LCC technique uses a feature fusion process comprising three DL models, namely residual network (ResNet), densely connected network (DenseNet), and Inception-ResNet-v2. Additionally, the DBO system can be employed for the optimum hyperparameter selection of the three DL approaches. For lung cancer detection purposes, the DBOMDFF-LCC system utilizes a long short-term memory (LSTM) system. The simulation result analysis of the DBOMDFF-LCC technique of the medical dataset is investigated using different evaluation metrics.

## 2. Related Works

Dhivya and Sharmila [16] proposed a multimodal method named Ensemble Deep Lung Disease Predictor (EDepLDP) architecture and developed a reliable solution for the quick recognition of different diseases using CXR and CT scans. Firstly, the images collected are segmented using U-Net architecture to obtain enhanced lung Regions of Interest (ROIs). Next, Xception and InceptionResNetV2 are used for hierarchically extracting informative features from segmented CXR scans. Yu et al. [17] developed a paediatric fine-grained diagnoses-assistant system to give precise and prompt diagnoses. This model has two phases: a disease identification stage and a test result structurization stage. The initial phase structuralizes the test outcomes by extracting numeric values from medical records, and the disease detection phase offers a diagnosis dependent upon text-form medical records and the structured information attained in the primary step. Agarwal et al. [18] developed a DL-based multilayer multimodal fusion system which emphasizes extracting the features of various layers and their combination. The disease detection method considered discriminative data from all the layers.

Behrad and Abadeh [19] developed one of the common multi-modalities, including fusion approaches and DL models. Also, the authors explained learning strategies such as end-to-end learning, multitask learning, and transfer learning. Next, the authors provided a summary of the DL method for a multi-modal medical data study. Ullah et al. [20] developed a strong DL model for the anatomical design in chest radiographs that exploits a dual encoded-decoded CNN. The pretrained encoded outcome is given as squeeze-and-excitation (SE) for increasing the representation power of the network. Wang et al. [21] developed and evaluated the efficiency of a DL architecture (3D-ResNet) dependent upon CT scans to differentiate nontuberculous mycobacterium lung disease (NTM-LD) in Mycobacterium TB lung disease (MTB-LD).

Akbulut [22] introduced a strong mechanism based on a new customized DL algorithm (ACL) that trained LSTM and attention models synchronously with the CNN model. The significant traces and stains in the CXR images are highlighted with the marker-controlled watershed (MCW) segmentation method. Moreover, the contribution of the strategy used in the presented method to classification accuracy was thoroughly assessed. Chouhan et al. [23] suggested a novel DL architecture for the diagnosis of pneumonia utilizing the TL model. Next, the authors developed an ensemble module which integrates output from each pretrained model that outperforms individual models, obtaining a remarkable performance in pneumonia detection.

Dalmaz et al. [24] presented a new approach dependent upon adversarial diffusion modeling, SynDiff, to enhance the efficiency of medical image translation. For capturing a direct connection of the image distribution, SynDiff leverages a conditional diffusion procedure which gradually maps the noise and source image onto the target image. Dalmaz et al. [25] proposed a novel generative adversarial approach for medical image synthesis, ResViT, that leverages the contextual sensitivity of vision transformers together with the precision of convolutional functions and realism of adversarial learning. The ResViT generator utilizes a central bottleneck containing a new aggregated residual transformer (ART) block which synergistically integrates residual convolution and transformer elements. Yurt et al. [26] examined a multi-stream system which aggregates data through several source images using a mixture of several one-to-one streams and joint many-to-one streams. The corresponding mapping features created in the one-to-one streams and shared mapping features created in the many-to-one stream were integrated with the fusion block.

## 3. The Proposed Model

**Figure 1:**
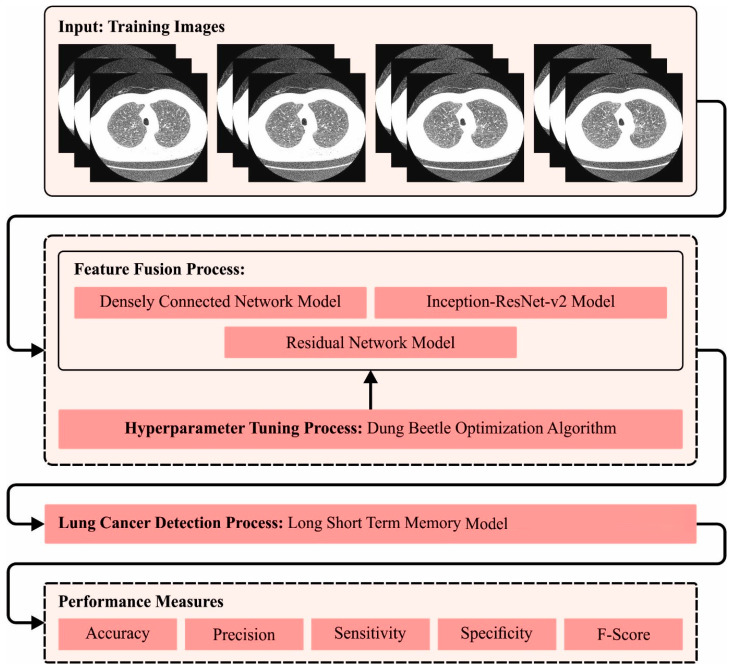
Overall flow of DBOMDFF-LCC system.

An automated lung cancer detection tool named the DBOMDFF-LCC system was established in this study. The aim of the projected DBOMDFF-LCC system is based on the feature fusion and hyperparameter tuning process. The DBOMDFF-LCC technique comprises three stage processes, namely feature fusion process, DBO-based hyperparameter selection, and LSTM classification. Figure 1 demonstrates the overall flow of the DBOMDFF-LCC system.

### 3.1. Feature Fusion Process

Primarily, the DBOMDFF-LCC technique uses a feature fusion process comprising three DL models, namely ResNet, DenseNet, and Inception-ResNet-v2. Entropy-based feature fusion is a procedure which integrates several features in distinct sources or modalities as a single feature representation utilizing the entropy model. The purpose is to capture complementary data in various features and improve the entire discriminative power of fused feature representations.

#### 3.1.1. ResNet

The ResNet18 model consists of five convolutional structures, an activation function (Softmax) layer, and a fully connected layer [27]. The initial Conv structure comprises an activation, 1D Conv, and BN layers. The complete parameters of this layer are as follows: the activation function of the activation layer utilized is ReLU, the number of Conv kernels from the 1D Conv layer was 64, the dimensional of Conv kernel is 7, and the padding mode remains unchanged. The second to fifth Conv structures had a similar form: they included a feature map block and Conv block; however, the count of Conv kernels differed based on the block. The numbers of Conv kernels of the second to fifth Conv designs are 64, 128, 256, and 512, respectively.

There were eight layers in all the Conv blocks: the BN layer, the activation layer, the 1D Conv layer, the 1-bit short-circuit linking layer, the 1D Conv layer, the activation layer, and the feature fusion layer. The parameter of the block was: ReLU can be exploited as an activation function of the activation layer, the Conv kernel size of the 1D Conv layer is fixed as three, and the padding mode remains unchanged. The mapping feature and Conv blocks have a similar infrastructure but are varied in the sense in which the 1D short-circuit linked layer has been altered for the mapping feature layer.

#### 3.1.2. DenseNet

The DenseNet201 structure has been trained primarily on ImageNet databases and contains three transition layers, four dense blocks, max-pooling, and convolutional layers [28]. The preceding layer was directly connected to the next layers from the network, which allows the mapping feature of the preceding layer that concatenated with the final layer, enhances the data flow among the layers, and permits the model to effectively extract and capture the gait features.

(1) $$f_{l}=H_{l}\left(\left[f_{0},\;f_{1},\;\ldots ,\;f{|}_{-1}\right]\right)$$

In Equation (1), l displays the layer and $\left[f_{0},\;f_{1,\;\ldots /}f_{l-1}\right]$ represents the feature concatenation. $H_{l}$ signifies the composite function which contains a 3×3 convolution function, BN, and ReLU activation. A dense block has been added as the model for adjusting the dimensional mapping features. The objective of the bottleneck layer is to diminish the count of input features that generate the network computational effect. The transition layer was inserted, then all the dense blocks except the final one were inserted to diminish the original size of mapping features by half. The transition layer carries out a 1×1 convolution layer and then 2×2 avg-pooling. The ability of every layer to add novel data to the network combined data is determined by the less growth rate.

#### 3.1.3. Inception-ResNet-v2

In the Inception-Resnet-v2 model, the pretrained topmost layer was previously removed since this model is highly particular to the trained rate [29]. This model utilizes the tricks and decisions of the Inception model with a residual connection variant. No preprocessing is conducted. First, the image was resized to 244×244, the input size for DCNN, and then resized to [0–1]. The resizing of images does not affect the shape of the cellular structure or the accuracy, and it permits lessening the computation rate. The topmost layer consists of a global average pooling layer, an FC layer of 256 neurons (with ReLU activation) and, lastly, the neuron that allows classification in the four classes (with Softmax activation). At an earlier stage, only the FC layer was trained. During the second stage, the DCNN was retrained on the topmost layer, and then finetuning of the weight of any pre-trained network layers was carried out. It is not uncommon to keep the weight of any bottom layers (caused by over-fitting issues) and only carry out the fine-tuning of high-level features. The most common features (blobs and edges) can thus be retained.

### 3.2. Hyperparameter Tuning Process

In this work, the DBO system can be employed for the optimum hyperparameter selection of the three DL approaches. The DBO is a recent swarm intelligence (SI) method based on dung beetle (DB) behaviors, namely dancing, ball rolling, stealing, breeding, foraging, and other activities, and the DBO method includes four optimization techniques: breeding, rolling balls, stealing, and foraging [30]. Unobstructed and obstructed modes are two behaviors of DB rolling.

#### 3.2.1. Obstacle-Free Mode

The DB exploits the sun in order to find direction in dung ball rolling once they move forward without any obstacles. In the DBO algorithm, as the light concentration changes, the location of the DB also changed as follows:

(2) $$x_{i}^{t+1}=x_{i}^{t}+a\times k\times x_{i}^{t-1}+b\times \left|x_{i}^{t}-x_{worst}^{t}\right|$$

In Equation (2), t denotes the number of the existing iterations, ke (0, 0.2] represents a set parameter signifying the flexure co-efficient, and $x_{i}^{t}$ represents the place of i-th DBs from the population at tth permutation. b denotes the invariant quantity within [0, 1], and α shows the natural co-efficient with the value of both [−1, 1], with −1 representing a deviation from the original direction and 1 signifying no deviation. $x_{worst}^{t}$ denotes the worst position from the existing specie, and the alteration in light concentration can be simulated using $|x_{i}^{t}-x_{worst}^{t}|.$

#### 3.2.2. Barrier Mode

The DB, once it meets an obstacle which prevents it from moving forward, desires to dance to recover a novel way forward. The author uses a tangent function to stimulate the dancing behaviors to attain the newest rolling direction that is only assumed from the range of [0, τc], and the beetle continues rolling the dung ball as soon as it finds a novel direction. The formula for upgrading the location:

(3) $$x_{i}^{t+1}=x_{i}^{t}+\tan\theta \left|x_{i}^{t}-x_{i}^{t-1}\right|$$

If θ=0, $\frac{\pi }{2},$ τπ, no changes occur in the location of DBs.

The female DB rolls the dung ball to a safer region for laying eggs and hides them to give a proper habitat for the progeny. The study presents a frontier option approach for modelling the brood ball position of a female DB:

(4) $$\left\{\begin{array}{l} Lf^{*}=max\{x_{gbest}^{t}\times (1-R),\;Lf\}\\ Uf^{*}=min\{x_{gbest}^{t}\times (1+R),\;Uf\} \end{array}\right.$$

In Equation (4), The lower and upper boundaries of the optimizer problems are Lf and Uf, respectively. $R=\frac{1-t}{T_{max}}$ and $T_{max}$ show the upper boundary of iterations. The existing population obtains the global optimal at $x_{gbest}^{t}$. The author defines the spawning’s lower and upper boundaries with Lf and Uf, which implies the position of DB spawn has been adjusted dynamically with iteration counts.

After a female DB finds the spawning region, she lays her eggs in that region. The region in which the location occurs is adjusted dynamically with the iteration counts; hence, the location of nestling spheres is dynamic in the iteration.

(5) $$B_{i}^{t+1}=x_{gbest}^{t}+b_{1}\times \left(B_{i}^{t}-Lf^{*}\right)+b_{2}\times \left(B_{i}^{t}-Uf^{*}\right)$$

In Equation (5), $B_{i}^{t+1}$ denotes the place of ith brood balls at the tth iterations, and D denotes the number of parameters in the optimization issues. $b_{1}\mathrm{and}$ $b_{2}$ characterize two arbitrary and independent vectors that have a D component and the location of nestling balls should be limited to the spawning region.

These behaviors are aimed mostly at smaller DBs. Some mature DBs emerge from the ground looking for food, and the optimum foraging region for smaller DBs is updated dynamically.

(6) $$\left\{\begin{array}{l} Lf^{l}=max\{x_{lbest}^{t}\times (1-R),\;Lf\}\\ Uf^{l}=min\{x_{lbest}^{t}\times (1+R),\;Uf\} \end{array}\right.$$

In Equation (6), R is similar to the prior definition, and $x_{lbest}^{t}$ signifies the location optimum location for the present population. The author uses $Lf^{l}$ and $Uf^{l}$ to determine the bottom and top bounds of the foraging area of lesser DBs, respectively. The position upgrade is given below:

(7) $$x_{i}^{t+1}=x_{i}^{t}+C_{1}\times \left(x_{i}^{t}-Lf^{l}\right)+C_{2}\times \left(x_{i}^{t}-Uf^{l}\right)$$

In Equation (7), $C_{1}$ is a number which follows a standard distribution while selected arbitrarily, as $C_{1}∼N(0,\;1)$, and $C_{2}$ shows the arbitrary vector within [0, 1] of 1×D.

During DB stealing, there exist any DBs that steal dung balls from other individuals, and the author updates the setting of thieving DBs:

(8) $$x_{i}^{t+1}=x_{lbest}^{t}+S\times g\times \left(\left|x_{i}^{t}-x_{gbest}^{t}\right|+\left|x_{i}^{t}-x_{lbest}^{t}\right|\right)$$

In Equation (8), S indicates a constant value and g denotes the vector of dimensional D that is selected arbitrarily, which obeys a standard distribution.

The DBO system progresses to a FF to accomplish better classifier results. It resolves a positive integer to exemplify the good effectiveness of candidate outcomes. During this study, the minimizing classifier error rate was supposed to be FF, as depicted in Equation (9).

(9) $$fitness\left(x_{i}\right)=Classifier\;Error\;Rate\left(x_{i}\right)=\frac{no.\;of\;misclassified\;instances}{Total\;no.\;of\;instances}*100$$

### 3.3. Lung Cancer Detection Process

To detect and classify lung cancer, the fused feature vectors are passed into the LSTM approach [31]. As the time interval rises, the recurrent HN approaches zero. This leads to the gradient diminishing a vulnerability that can be encountered while applying RNN for long-term data sequence modeling. The memory cell has a node connected with the recurrent edge of a set weighted node, thus guaranteeing that the gradient survives a longer time step without vanishing. The multiplicative gate allows the model to store data over a longer period, thus removing the gradient vanishing problem usually observed in traditional NN models.

Assume input sequence data are represented as x= $x_{1}+x_{2}+x_{3},\ldots ,x_{t}$ and output series data are represented as $y=y_{1}+y_{2}+y_{3},\ldots ,y_{t}$, where τ denotes the forecast horizon. The LSTM calculates the forecast outcome automatically in the next time step using the prior data, without predefining the lag observation to utilize:

(10) $$i_{t}=\sigma \left(W_{xi}x_{t}+W_{hi}h_{t-1}+W_{ci}c_{t-1}+b_{i}\right)$$

(11) $$f_{t}=\sigma \left(W_{xf}x_{t}+W_{hf}h_{t-1}+W_{cf}c_{t-1}+b_{f}\right)$$

(12) $$c_{t}=f_{t}c_{t-1}+i_{t}g\left(W_{xc}x_{t}+W_{hc}h_{t-1}+b_{c}\right)$$

(13) $$o_{t}=\sigma \left(W_{xo}x_{t}+W_{ho}h_{t-1}+W_{co}c_{t}+b_{o}\right)$$

(14) $$h_{t}=o_{t}h\left(c_{t}\right)$$

where 0 represents a standard logistic sigmoid function and W and b illustrate the weighted matrix and bias vector, respectively, defined as:

(15) $$\sigma \left(x\right)=\frac{1}{1+e^{-x}}$$

(16) $$g\left(x\right)=\frac{4}{1+e^{-x}}-2$$

(17) $$h\left(x\right)=\frac{2}{1+x}-1$$

where the parameters c i,f, and o indicate the cell activation vector, input gate, forget gate, and output gate, respectively. g(.) and h(.) denote the respective transformations of the sigmoid function. This certain feature makes LSTM an accurate and reliable method for lung cancer detection.

## 4. Experimental Validation

**Figure 2:**
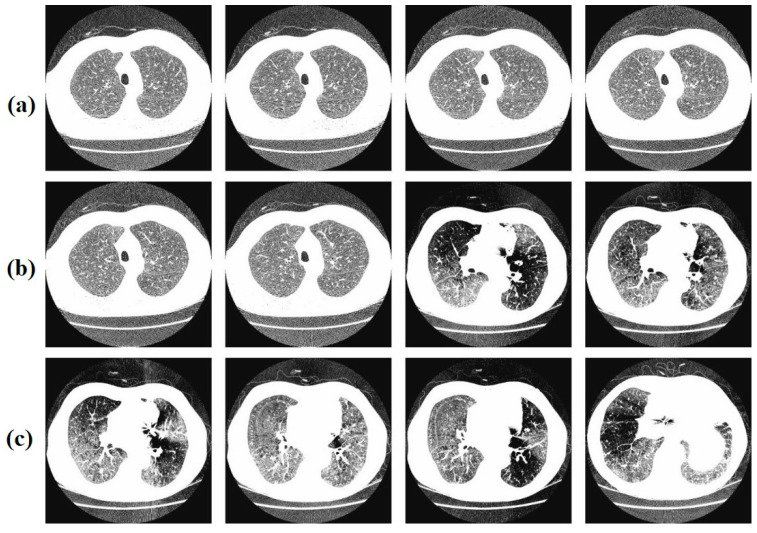
Sample images. (**a**) Normal, (**b**) benign, (**c**) malignant [32].

In this section, the results of the DBOMDFF-LCC approach are examined on the lungdb database [32], comprising 100 samples and 3 classes, as demonstrated in Table 1. Figure 2 represents the sample images. For experimental validation, 80:20 and 70:30 of training/testing dataset is used.

**Figure 3:**
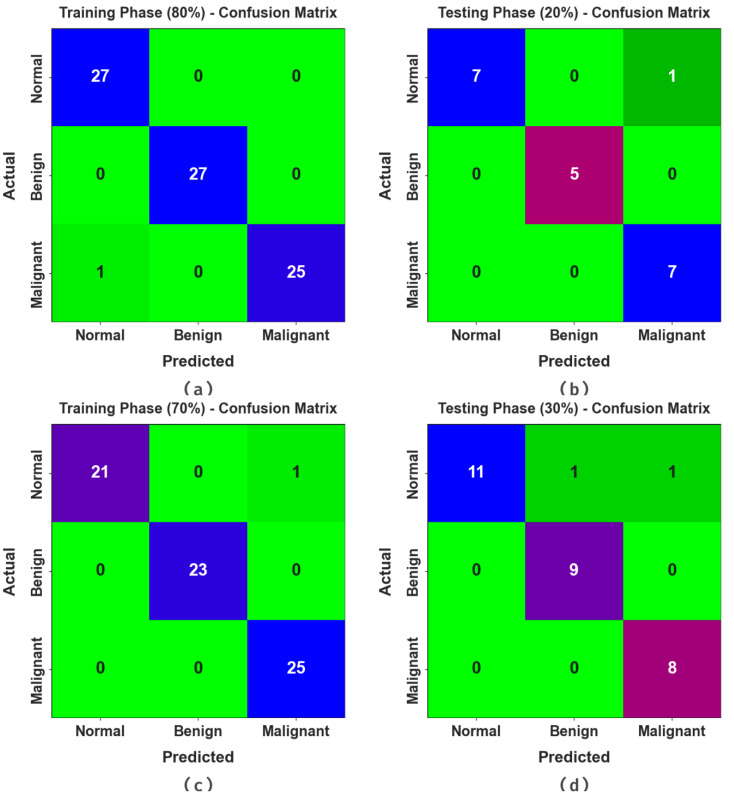
Confusion matrices of DBOMDFF-LCC system. (**a**,**b**) 80:20 of TRP/TSP and (**c**,**d**) 70:30 of TRP/TSP.

The confusion matrices of the DBOMDFF-LCC approach to the lung cancer recognition process are demonstrated in Figure 3. The outcomes stated that the DBOMDFF-LCC system recognizes three classes proficiently.

**Figure 4:**
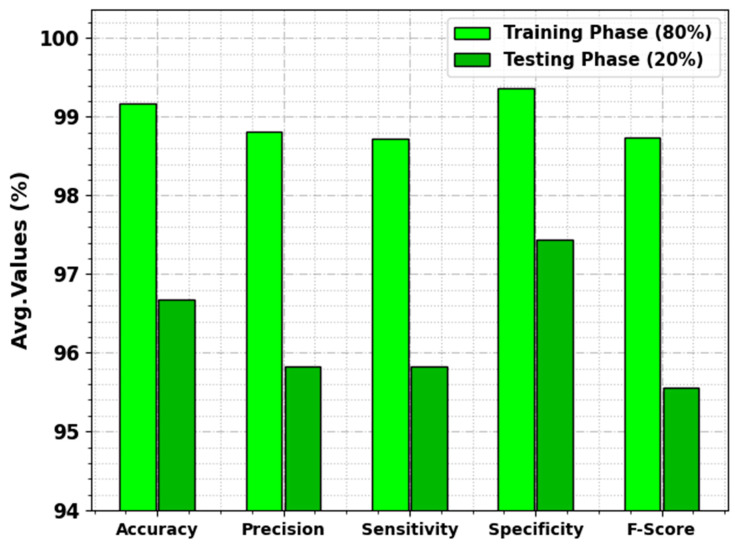
Average outcome of DBOMDFF-LCC approach on 80:20 of TRP/TSP.

In Table 2 and Figure 4, the overall lung cancer detection results of the DBOMDFF-LCC technique are exemplified on 80:20 of TRP/TSP. The outcomes exhibit that the DBOMDFF-LCC system recognizes all three classes efficiently. For samples with 80% of TRP, the DBOMDFF-LCC system gains average $accu_{y}$, $prec_{n}$, $sens_{y}$, $spec_{y}$, and $F_{score}$ of 99.17%, 98.81%, 98.72%, 99.37%, and 98.74%, respectively. Also, with 20% of TSP, the DBOMDFF-LCC method reaches average $accu_{y}$, $prec_{n}$, $sens_{y}$, $spec_{y}$, and $F_{score}$ of 96.67%, 95.83%, 95.83%, 97.44%, and 95.56%, respectively.

**Figure 5:**
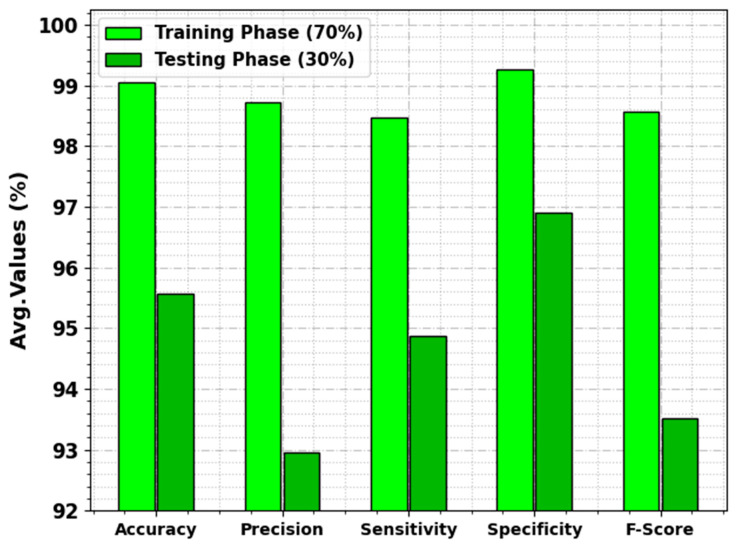
Average outcome of DBOMDFF-LCC system on 70:30 of TRP/TSP.

In Table 3 and Figure 5, the overall lung cancer detection results of the DBOMDFF-LCC system are demonstrated on 70:30 of TRP/TSP. The outcome exhibited that the DBOMDFF-LCC system recognizes all three classes efficiently. For instance, with 70% of TRP, the DBOMDFF-LCC method reaches average $accu_{y}$, $prec_{n}$, $sens_{y}$, $spec_{y}$, and $F_{score}$ of 99.05%, 98.72%, 98.48%, 99.26%, and 98.57%, respectively. In addition, with 30% of TSP, the DBOMDFF-LCC approach attains an average $accu_{y}$, $prec_{n}$, $sens_{y}$, $spec_{y}$, and $F_{score}$ of 95.56%, 92.96%, 94.87%, 96.90%, and 93.51%, respectively.

**Figure 6:**
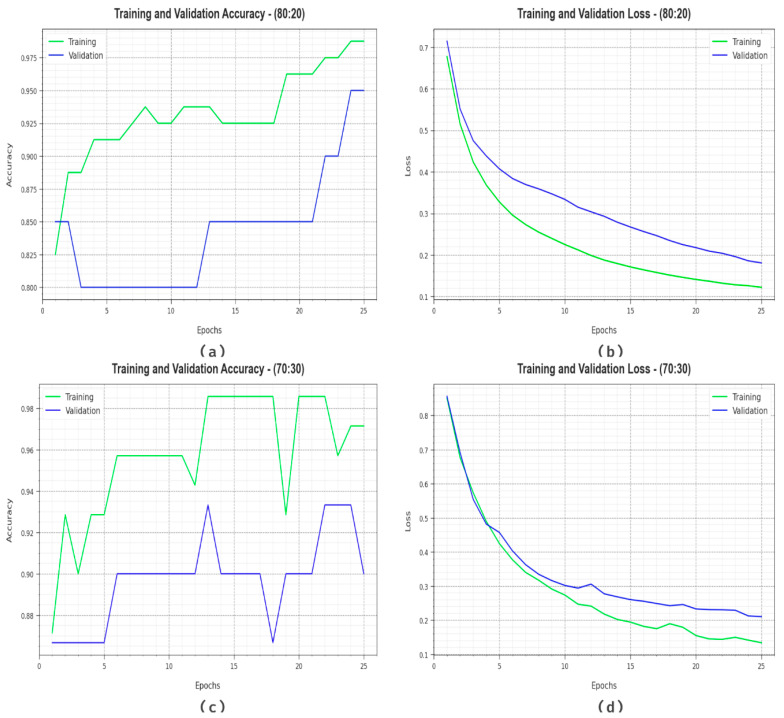
(**a**,**c**) Accuracy curve on 80:20/70:30 and (**b**,**d**) loss curve on 80:20/70:30.

Figure 6 demonstrates the classifier outcome of the DBOMDFF-LCC method on 80:20/70:30. Figure 6a,c demonstrates the accuracy examination of the DBOMDFF-LCC model on 80:20/70:30. The result stated that the DBOMDFF-LCC technique attains maximum accuracy values over higher epochs. In addition, the higher validation accuracy over training accuracy illustrates that the DBOMDFF-LCC method learns capably on the test database. Finally, Figure 6b,d illuminates the loss examination of the DBOMDFF-LCC approach on 80:20/70:30. The outcome implied that the DBOMDFF-LCC approach gains adjacent values of training and validation loss. The DBOMDFF-LCC system learns effectively on the test database.

**Figure 7:**
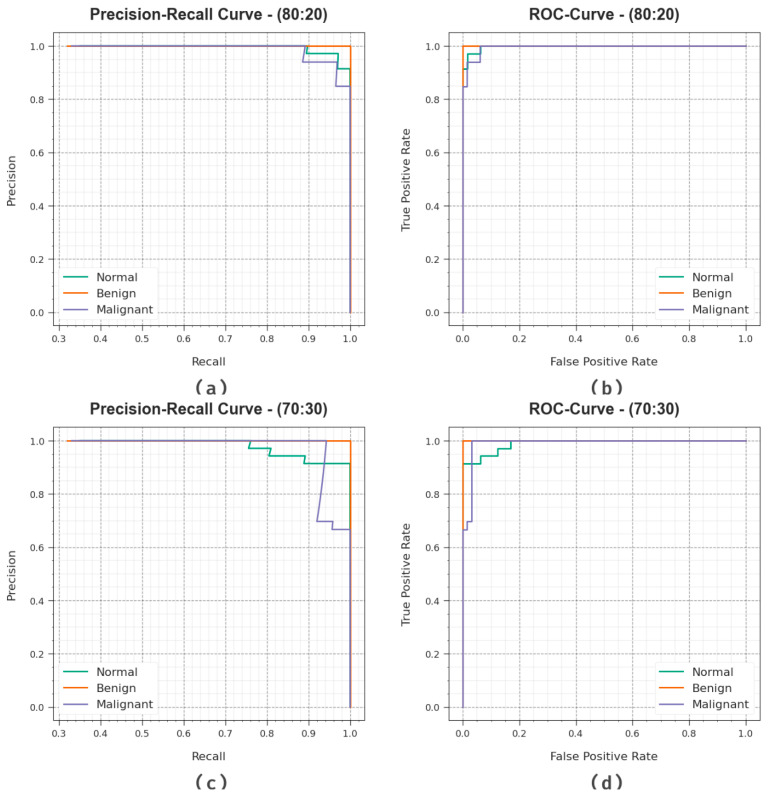
(**a**,**c**) PR curve on 80:20/70:30 and (**b**,**d**) ROC curve on 80:20/70:30.

Figure 7 demonstrates the classifier results of the DBOMDFF-LCC algorithm at 80:20/70:30. Figure 7a,c establishes the PR examination of the DBOMDFF-LCC approach on 80:20/70:30. The results implied that the DBOMDFF-LCC technique results in superior values of PR. In addition, it is clear that the DBOMDFF-LCC methodology can reach higher PR values in all classes. Lastly, Figure 7b,d illustrates the ROC examination of the DBOMDFF-LCC model under 80:20/70:30. The outcome implied that the DBOMDFF-LCC system resulted in improved ROC values. Also, the DBOMDFF-LCC method can extend enhanced ROC values on all classes.

**Figure 8:**
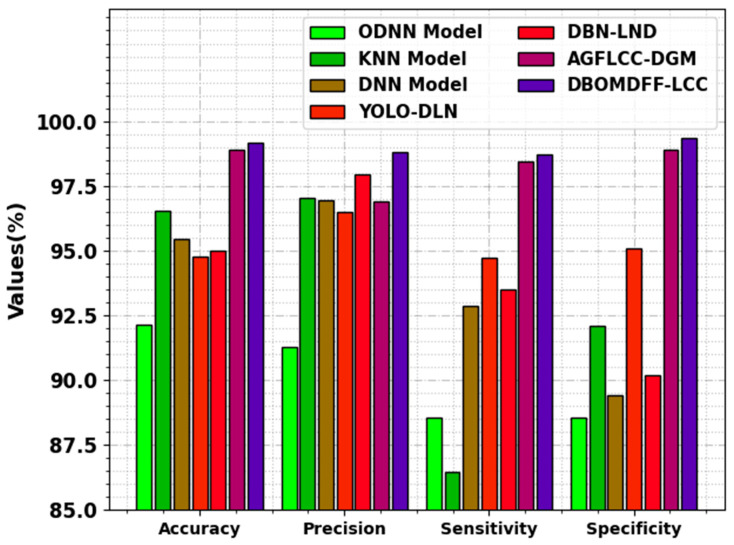
Comparative outcome of DBOMDFF-LCC system with other approaches.

In Table 4 and Figure 8, a comparison result of the DBOMDFF-LCC method is offered with existing systems [33]. The outcome highlighted that the DBOMDFF-LCC approach reaches enhanced results. Based on $accu_{y}$, the DBOMDFF-LCC technique obtains a higher $accu_{y}$ of 99.17%, while the ODNN, KNN, DNN, YOLO-DLN, DBN-LND, and AGFLCC-DGM models accomplish a lower $accu_{y}$ of 92.12%, 96.52%, 95.45%, 94.75%, 95%, and 98.91%, respectively. Meanwhile, based on $prec_{n}$, the DBOMDFF-LCC approach gains a superior $prec_{n}$ of 98.81%, while the ODNN, KNN, DNN, YOLO-DLN, DBN-LND, and AGFLCC-DGM approaches achieve a lesser $prec_{n}$ of 91.29%, 97.03%, 96.95%, 96.49%, 97.92%, and 96.88%, respectively. Furthermore, with respect to $sens_{y}$, the DBOMDFF-LCC technique obtains a higher $sens_{y}$ of 98.72%, while the ODNN, KNN, DNN, YOLO-DLN, DBN-LND, and AGFLCC-DGM systems accomplish a lower $sens_{y}$ of 88.56%, 86.45%, 92.85%, 94.70%, 93.50%, and 98.46%, respectively. These results show the maximum lung cancer detection efficiency of the DBOMDFF-LCC technique. The enhanced performance of the proposed model is due to the feature fusion and hyperparameter tuning process.

## 5. Conclusions

An automated lung cancer detection tool named DBOMDFF-LCC system was established in this study. The aim of the projected DBOMDFF-LCC algorithm is based on the feature fusion and hyperparameter tuning process. Primarily, the DBOMDFF-LCC technique uses a feature fusion process comprising three DL models, namely ResNet, DenseNet, and Inception-ResNet-v2. Additionally, the DBO system was employed for the optimum hyperparameter selection of the three DL algorithms. For lung cancer detection purposes, the DBOMDFF-LCC technique utilized the LSTM approach. The simulation result analysis of the DBOMDFF-LCC system on the medical dataset is investigated using different evaluation metrics. The extensive comparative results highlighted the betterment of the DBOMDFF-LCC technique of lung cancer classification.

## Figures and Tables

**Table 1:** Details of databases.

Class	No. of Samples
Normal	35
Benign	32
Malignant	33
Total Samples	100

**Table 2:** Lung cancer detection outcome of DBOMDFF-LCC approach on 80:20 of TRP/TSP.

Class	$Accu_{y}$	$Prec_{n}$	$Sens_{y}$	$Spec_{y}$	$F_{Score}$
Training Phase (80%)					
Normal	98.75	96.43	100.00	98.11	98.18
Benign	100.00	100.00	100.00	100.00	100.00
Malignant	98.75	100.00	96.15	100.00	98.04
Average	99.17	98.81	98.72	99.37	98.74
Testing Phase (20%)					
Normal	95.00	100.00	87.50	100.00	93.33
Benign	100.00	100.00	100.00	100.00	100.00
Malignant	95.00	87.50	100.00	92.31	93.33
Average	96.67	95.83	95.83	97.44	95.56

**Table 3:** Lung cancer detection outcome of DBOMDFF-LCC system on 70:30 of TRP/TSP.

Class	$Accu_{y}$	$Prec_{n}$	$Sens_{y}$	$Spec_{y}$	$F_{Score}$
Training Phase (70%)					
Normal	98.57	100.00	95.45	100.00	97.67
Benign	100.00	100.00	100.00	100.00	100.00
Malignant	98.57	96.15	100.00	97.78	98.04
Average	99.05	98.72	98.48	99.26	98.57
Testing Phase (30%)					
Normal	93.33	100.00	84.62	100.00	91.67
Benign	96.67	90.00	100.00	95.24	94.74
Malignant	96.67	88.89	100.00	95.45	94.12
Average	95.56	92.96	94.87	96.90	93.51

**Table 4:** Comparative outcome of DBOMDFF-LCC algorithm with other approaches [33].

Methods	$Accu_{y}$	$Prec_{n}$	$Sens_{y}$	$Spec_{y}$
ODNN Model	92.12	91.29	88.56	88.54
KNN Model	96.52	97.03	86.45	92.10
DNN Model	95.45	96.95	92.85	89.40
YOLO-DLN	94.75	96.49	94.70	95.10
DBN-LND	95.00	97.92	93.50	90.20
AGFLCC-DGM	98.91	96.88	98.46	98.89
DBOMDFF-LCC	99.17	98.81	98.72	99.37

## Data Availability

The data presented in this study are available in this article.
